# Single cell transcriptomics of mouse kidney transplants reveals a myeloid cell pathway for transplant rejection

**DOI:** 10.1172/jci.insight.141321

**Published:** 2020-10-15

**Authors:** Anil Dangi, Naveen R. Natesh, Irma Husain, Zhicheng Ji, Laura Barisoni, Jean Kwun, Xiling Shen, Edward B. Thorp, Xunrong Luo

**Affiliations:** 1 Division of Nephrology, Department of Medicine, Duke University School of Medicine, Durham, North Carolina, USA.; 2 Department of Biomedical Engineering, Duke University Pratt School of Engineering, Durham, North Carolina, USA.; 3 Department of Biostatistics & Bioinformatics,; 4 Department of Pathology,; 5 Department of Surgery, and; 6 Duke Transplant Center, Duke University School of Medicine, Durham, North Carolina, USA.; 7 Department of Pathology, Northwestern University Feinberg School of Medicine, Chicago, Illinois, USA.

**Keywords:** Immunology, Transplantation, Bioinformatics, Macrophages, Organ transplantation

## Abstract

Myeloid cells are increasingly recognized as major players in transplant rejection. Here, we used a murine kidney transplantation model and single cell transcriptomics to dissect the contribution of myeloid cell subsets and their potential signaling pathways to kidney transplant rejection. Using a variety of bioinformatic techniques, including machine learning, we demonstrate that kidney allograft–infiltrating myeloid cells followed a trajectory of differentiation from monocytes to proinflammatory macrophages, and they exhibited distinct interactions with kidney allograft parenchymal cells. While this process correlated with a unique pattern of myeloid cell transcripts, a top gene identified was *Axl*, a member of the receptor tyrosine kinase family Tyro3/Axl/Mertk (TAM). Using kidney transplant recipients with *Axl* gene deficiency, we further demonstrate that Axl augmented intragraft differentiation of proinflammatory macrophages, likely via its effect on the transcription factor Cebpb. This, in turn, promoted intragraft recruitment, differentiation, and proliferation of donor-specific T cells, and it enhanced early allograft inflammation evidenced by histology. We conclude that myeloid cell *Axl* expression identified by single cell transcriptomics of kidney allografts in our study plays a major role in promoting intragraft myeloid cell and T cell differentiation, and it presents a potentially novel therapeutic target for controlling kidney allograft rejection and improving kidney allograft survival.

## Abstract 2

In a murine model of allogeneic kidney transplantation, single-cell transcriptomics identifies that myeloid cell Axl expression promotes allograft rejection by inducing inflammatory macrophage differentiation.

## Introduction

Adaptive immunity focusing on T cell biology has dominated studies of transplant rejection in the past several decades. However, innate immunity has recently become recognized as an important player in transplantation (1–4), demonstrating previously unidentified roles in promoting ischemic reperfusion injuries (5), priming adaptive immune responses (1, 6), or perpetuating chronic rejection (7). Among innate immune cells, tissue macrophages have been shown to influence organ homeostasis and defense (8). Specifically, kidney macrophages have been characterized both transcriptionally (9) and functionally (10, 11), and they are implicated in numerous kidney disease processes, including nephritis (12), acute kidney injury (13), and kidney fibrosis (14). It has also been shown that, during an episode of acute cell–mediated rejection, kidney allografts are typically heavily infiltrated with macrophages in addition to T lymphocytes (15, 16) — a finding corroborated by the association of macrophage and dendritic cell–specific transcriptomes with T cell–mediated rejection (17). These data, thus, suggest a potential role of intragraft macrophages in shaping the host immune response to the transplanted kidneys (18).

To this point, most studies of innate immunity in transplantation have relied on specific cellular and genetic tools to test a priori hypotheses in transplantation models. However, recent developments in the single cell RNA sequencing (scRNA-seq) technology (19, 20) have made it possible to simultaneously interrogate, in parallel and in an unbiased fashion, cellular signaling and function at individual cell levels and, additionally, to identify potentially novel cell-to-cell interactions through profiling receptor and ligand transcriptomics of individual cells (21, 22). Armed with this technology, we examined distinct innate cell-specific transcriptomes present in rejecting versus tolerized kidney allografts in a murine allogeneic kidney transplant model. For transplantation tolerance induction, we used an established experimental regimen in which robust donor-specific tolerance is induced by peritransplant recipient infusions of donor splenocytes (SP) treated with a chemical cross-linker ethylenecarbodiimide (ECDI-SP) (23, 24). Applying scRNA-seq to this model, we identified myeloid cell transcripts, myeloid cell–kidney cell interactions, and intragraft myeloid cell differentiation trajectories that distinguish kidney allograft rejection from tolerance.

Among the top genes identified in our study was the receptor tyrosine kinase (RTK) Axl, a member of the cell surface RTK family Tyro3/Axl/Mertk (TAM) (25). Existing literature points to inflammation resolution as a major function of Axl (26). Consequently, *Axl* gene deficiency, in combination with *Mertk* deficiency, leads to autoimmunity (26). However, little is known of the role of Axl in transplantation. To our surprise, guided by our findings from scRNA-seq of transplanted kidneys, we observed that recipient myeloid cell *Axl* expression, in fact, played a critical role in the priming and trafficking of donor-specific T cells to the kidney allograft. Consequently, recipient *Axl* deficiency or Axl inhibition significantly reduced early kidney allograft inflammation, predicting superior long-term allograft function.

## Results

### scRNA-seq identifies functionally distinct immune and parenchymal populations in kidney allografts.

BALB/c kidneys were transplanted into fully MHC-mismatched bilaterally nephrectomized Black 6 (B6 or C57BL/6J) recipients. Untreated recipients promptly rejected the kidney allograft (rejecting group), whereas BALB/c ECDI-SP–treated recipients developed graft tolerance (tolerized group), as we previously described (27). Transplanted kidneys from both groups were collected on posttransplant day 15 (d15). Naive untransplanted kidneys (naive group) were obtained on the same days as controls. We sequenced a total of 6 kidneys with 2 kidneys from each group.

**Figure 1:**
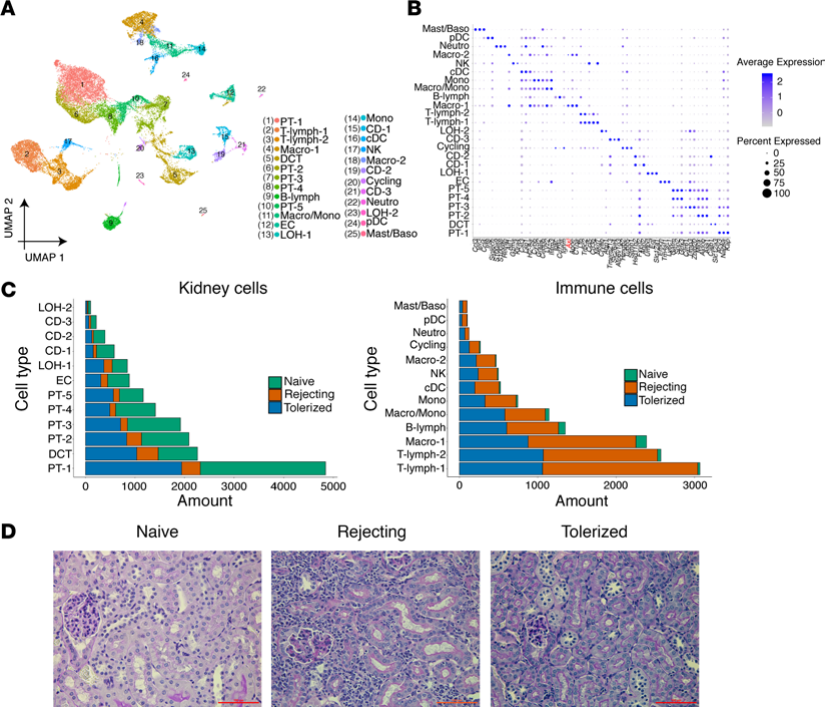
Distinct kidney and immune cell clusters are identified by scRNA-seq of kidney allografts. (**A**) UMAP of 25 cell clusters identified from combined single cells sequenced from rejecting, tolerized kidney allografts, and naive untransplanted kidneys (*n* = 2 each, total 6 kidneys). A total of 30,053 cells were represented in the UMAP. (**B**) Dot plot illustrating each cell cluster and their expression of selected marker genes. (**C**) Bar graphs showing the number of cells in each kidney or immune cell cluster by condition (naive, rejecting, or tolerized). (**D**) Representative photomicrographs showing histopathology (by PAS staining) of the kidneys used for scRNA-seq analysis (representative of *n* = 2 in each condition). Scale bar: 100 μm. PT, proximal tubule; T-lymph, T lymphocyte; Macro, macrophage; DCT, distal convoluted tubule; B-lymph, B lymphocyte; Macro/Mono, macrophage/monocyte; EC, endothelial cell; LOH, Loop of Henle; CD, collecting duct; cDC, conventional DC; Neutro, neutrophil; pDC, plasmacytoid DC; Mast/Baso, mast cell/basophil; UMAP, Uniform Manifold Approximation and Projection.

In approaching the problem of identifying discrete cell populations comprising this full data set, the nature of samples and further stress imputed through cell dissociation presented the first concern. Specifically, cells from both transplanted conditions would be subjected to inflammatory stress, and this would manifest in the data as many mitochondrial genes being mapped per cell due to prelysed cells, as well as a higher number of expected doublets (28). Therefore, we first filtered out low-quality cells on the basis of low unique molecular identifier (UMI) values and high mitochondrial RNA content. We then applied the bioinformatic tool DoubletDecon (29) to confidently remove doublets from our data set. Using this more biologically realistic data set, we performed normalization, scaling, and clustering with Seurat (30). We identified 25 clusters of conserved cell types across naive, rejecting, and tolerized kidneys from a total of approximately 30,000 cells sequenced (Figure 1A and Supplemental Figure 1; supplemental material available online with this article; https://doi.org/10.1172/jci.insight.141321DS1). Of the 25 clusters, 12 were identified as kidney cell types. Thirteen were identified as immune cell types, including 1 cluster of cycling cells based on their large number of highly expressed cell cycle–related genes.

Each cluster of cells was defined by a set of unique genes predominantly expressed only by that cluster in comparison with all other clusters combined (cluster-defining genes; Figure 1B). The cluster-defining genes of immune cell types aligned well with the ImmGen database (31), and those of kidney cell types aligned with classical marker genes from published literature (21) (Figure 1B). At the level of total cell numbers, we found that tolerized and naive kidneys had a significantly higher number of cells in all kidney cell clusters than in rejecting kidneys (Figure 1C, left panel), supporting that, at this time point, tolerized kidneys preserved their kidney function similar to naive kidneys, whereas rejecting kidneys suffered from loss of kidney function. In contrast, the number of cells in all immune cell clusters was the highest in rejecting kidneys followed by tolerized kidneys, and it was markedly lower and often negligible in naive kidneys (Figure 1C, right panel). This observation was further confirmed by histological examination of the kidneys in these groups (Figure 1D), in which immune cell infiltration was most aggressive in rejecting kidneys and almost completely absent in naive kidneys, with tolerized kidneys manifesting an intermediate level of immune cell infiltration. The presence in tolerized versus rejecting kidneys of a relatively comparable number of immune cells but significantly disparate number of kidney cells led us to hypothesize that immune cells in tolerized kidneys were functionally distinct from those in rejecting kidneys, thus interacting with kidney cells differently at the molecular level to promote suppression of antidonor immune responses. This hypothesis was the rationale for the later ligand-receptor (LR) analysis presented in this study (see below).

### Specific genes are differentially expressed in rejecting versus tolerized kidney allografts.

Next, we examined differentially expressed genes (DEGs) in rejecting versus tolerized kidneys. First, we focused on kidney cell clusters. Naive untransplanted kidneys were used as controls. We noted that genes upregulated in tolerized kidneys tended to be similarly upregulated in naive untransplanted kidneys but not in rejecting kidneys. Two such examples are shown in Supplemental Figure 2A: expressions of both *Kap* and *Gpx3* were consistently enriched across all tubular segments of the nephron in tolerized (blue) as in naive (green) kidneys, but not in rejecting (red) kidneys. Both genes are involved in cell metabolism and responses to inflammation (32, 33). Additionally, genes involved in canonical kidney function such as *Atp5k* and *Miox* were similarly enriched in tolerized and naive kidneys, but they were diminished in rejecting kidneys (Supplemental Figure 2A). These findings supported that sustaining a close to normal kidney function is an integral component of the tolerized phenotype. Several genes involved in immune regulation, such as *Egf* (34) and *S100g* (35), also showed significantly heightened expression in kidney cells from tolerized and naive kidneys in comparison with those from rejecting kidneys (Supplemental Figure 2B). In contrast, the expression of chemokine genes such as *Ccl5* and *Ccl8* was significantly enhanced in kidney cells from rejecting kidneys in comparison with those from tolerized or naive kidneys (Supplemental Figure 2C).

Next, we examined DEGs in immune cell clusters from rejecting versus tolerized kidneys. We first verified that T lymphocyte immune responses were inhibited in tolerized kidneys, as we previously published (23, 24). A large array of T cell genes was found to be expressed at a higher level and/or in a higher number of cells in rejecting kidneys in comparison with tolerized kidneys. Expressions of representative genes in T cells such as *granzyme B*, *IL-21R*, *TNF-α*, and *TNF-α–induced protein 3* in rejecting versus tolerized kidneys are shown as UMAPs in Supplemental Figure 3. Among these, violin plots of the expression of *IL-21R* and *granzyme B* in the T-lymph–1 cluster cells are representatively shown in Supplemental Figure 4A. On the other hand, several genes were found to be significantly upregulated in T cells from tolerized kidneys, an example being the IgE receptor–related gene *Fcer1g* (Supplemental Figure 4B). This gene has previously been implicated in asthma and allergic reactions (36, 37); however, its role in T cell immune tolerance in transplantation remains unknown.

**Figure 2:**
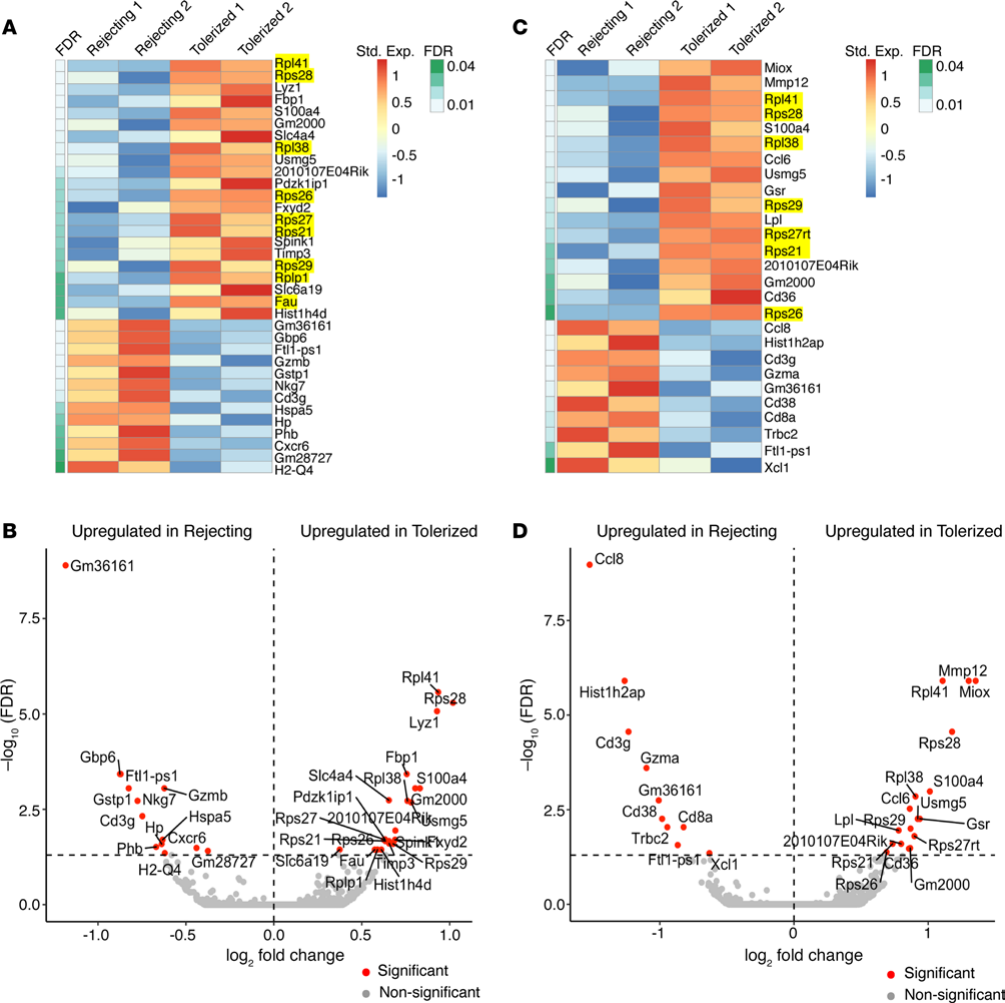
Specific genes are differentially expressed by myeloid cell clusters from rejecting versus tolerized kidney allografts. (**A**) The heatmap showing the standardized expression values of all significantly differential genes (FDR < 0.05) in the Macro-1 cell cluster from rejecting versus tolerized allografts. Each row represents a gene, and each column represents a pseudobulk sample by pooling Macro-1 cells for each rejecting or tolerized sample. Normalized expression values and FDR were obtained using DESeq2. The expression values were standardized for each gene across all pseudobulk samples. (**B**) Volcano plot for the Macro-1 cell cluster showing the log_2_ fold change (*x* axis) and –log_10_(FDR) (*y* axis) of the differential analysis. Each dot represents a gene. The dashed horizontal gray line represents an FDR value of 0.05. Significant genes (FDR < 0.05) are marked as red, and other genes are marked as gray. Names of the significant genes are displayed alongside the dots. (**C**) The heatmap of relative expression of DEGs in the Macro/Mono cell cluster from rejecting versus tolerized kidney allografts similarly generated as in **A**. Highlighted genes in **A** and **C** are representative of a ribosomal protein gene signature. (**D**) Volcano plot for the Macro/Mono cell cluster similarly generated as in **B**.

Lastly, we examined DEGs in myeloid cell clusters from rejecting versus tolerized kidneys. Heatmaps and volcano plots of the 2 largest clusters, namely Macro-1 and Macro/Mono, are shown in Figure 2, A–D. Interestingly, we found a significant number of ribosomal protein genes (highlighted in yellow in the heatmaps for the Macro-1 and Macro/Mono cell clusters) that were consistently more highly expressed in tolerized kidneys than in rejecting kidneys (Figure 2, A and C). These ribosomal proteins are known to respond to stress and regulate cell proliferation (38). In addition to ribosomal protein genes, genes conventionally known to be implicated in myeloid cell function also showed differential expression between rejecting and tolerized conditions. UMAPs of representative genes including *Cxcr6*, *Xcl1*, and *Ccl8*, are shown in Supplemental Figure 5.

Collectively, our findings suggest that numerous DEGs in both kidney cells as well as immune cells are implicated in the differential phenotype of rejection versus tolerance of the kidney allograft and that ribosomal proteins may play significant roles in modulating immune responses via myeloid cells.

### LR analysis reveals potential cell-to-cell interactions, distinguishing kidney allograft rejection versus tolerance.

**Figure 3:**
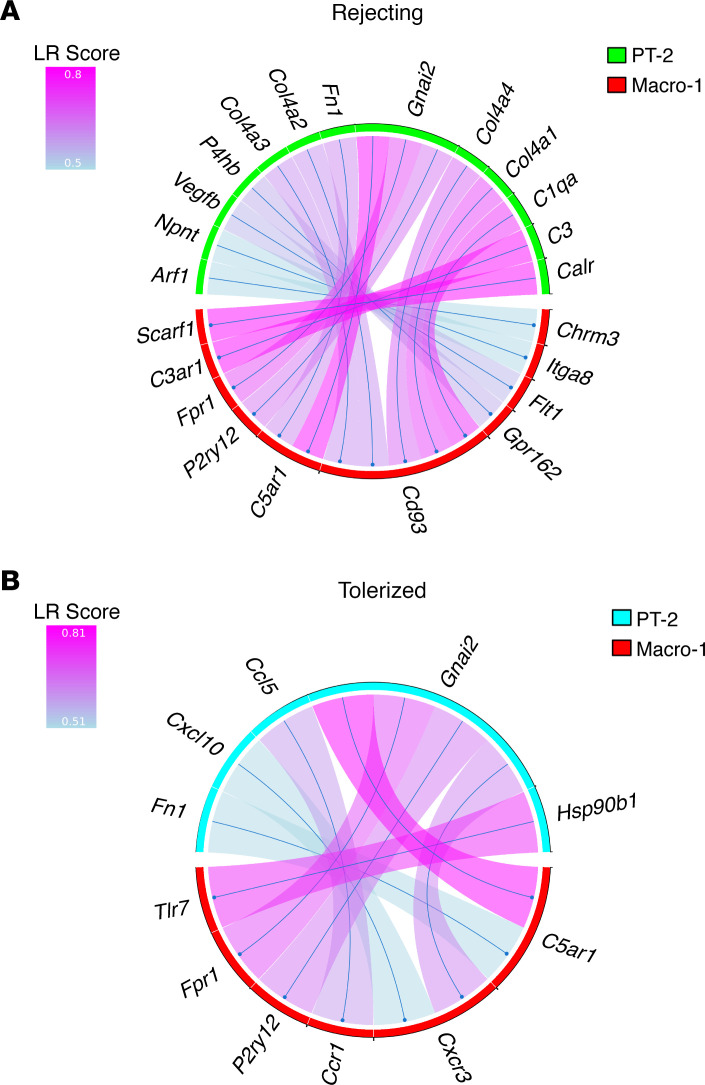
Distinct ligand-receptor (LR) interactions are present between Macro-1 and PT-2 cell clusters in rejecting versus tolerized kidney allografts. (**A**) LR interactions between Macro-1 and PT-2 cell clusters in rejecting kidneys. (**B**) LR interactions between Macro-1 and PT-2 cell clusters in tolerized kidneys.

As mentioned previously, data in Figure 1C suggest that, in tolerized kidneys, immune cells interacted with kidney cells differently from those in rejecting kidneys, leading to different outcomes of antidonor immune responses in these kidneys. To test this hypothesis, we investigated potential LR interactions between immune cells and kidney cells in rejecting versus tolerized kidney allografts. Using SingleCellSignalR (39), a bioinformatic tool that identifies potential LR pairs expressed by cluster pairs, we identified LR interactions between all individual cell clusters (Supplemental Table 1, Supplemental Table 2, Supplemental Table 3). Among these, of particular interest were LR interactions between the Macro-1 cluster cells and the proximal tubule (PT-2) cluster cells (Figure 3). First, the LR pairs between these 2 cell clusters were more numerous in rejecting than in tolerized kidneys, and many pairs were unique to the specific phenotype (rejection versus tolerance). Specifically, the majority of interactions identified in the rejecting kidneys (Figure 3A) were related to complement-mediated myeloid cell immune responses, such as interactions between ligands C1qa, Col4a1, and Col4a4 and their cognate receptor CD93. The lectin receptor CD93 is a complement receptor implicated in phagocytosis, intercellular adhesion, and leukocyte extravasation (40, 41); its expression in myeloid cells is only relevant when its ligands are also expressed, in this case, by kidney cells under stress. The presence of CD93-mediated LR pairs in rejecting kidneys suggested that it may serve as a potentially novel myeloid cell receptor that mediated inflammatory interactions between myeloid cells and tubular cells. Other prominent complement-related LR pairs unique to rejecting kidneys included C3-C3ar1 and Cair-Scarf1, the latter implicated in complement-mediated apoptotic cell clearance and innate immunity (42). In tolerized kidneys (Figure 3B), however, we found Hsp90b1 and Tlr7 as a unique LR pair, indicating that the interaction between TLR7 and the TLR chaperone Hsp90b1 may be necessary for TLR7-mediated tolerance as previously described (43). There were also common LR pairs in both rejecting and tolerized kidneys. Notably, Gnai2 pairing with C5ar1, Fpr1, and P2ry12 was found in both phenotypes (Figure 3, A and B), suggesting their role in maintaining canonical myeloid cell functions (44).

Collectively, these findings suggest that unique LR pairs between myeloid cells and kidney cells distinguish rejecting versus tolerized kidneys in our model. Whether such interactions represent a cause or a consequence of the respective phenotype will be of considerable interest for future studies.

### RNA Velocity and monocle pseudotime analyses reveal potential paths of kidney allograft–infiltrating myeloid cell differentiation.

In addition to cell clustering and identification of DEGs, a unique advantage of scRNA-seq data is its ability to be used in various inferential algorithms to predict future cell states. RNA Velocity (45) is one such algorithm. It computes the numerical differences in spliced and unspliced mRNA for every gene in a cell, which are then used to compute the future amount of mRNA in a cell. Since quantitation of particular mRNA species in a cell can be a proxy for the identity of that cell, RNA Velocity of scRNA-seq data can be used to predict evolution trajectories between cell populations on a time scale.

**Figure 4:**
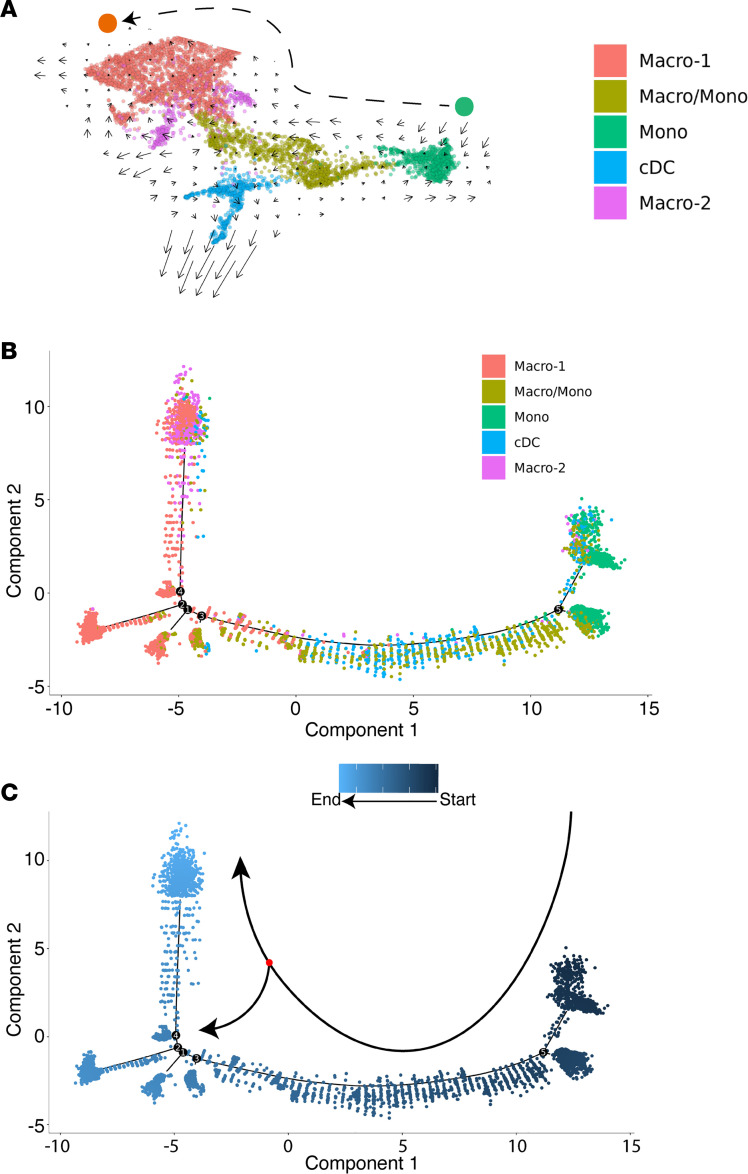
RNA Velocity and Monocle pseudotime analyses reveal potential paths of kidney allograft–infiltrating myeloid cell differentiation. (**A**) RNA Velocity analysis of the 5 myeloid cell clusters depicts a differentiation path from the Mono cell cluster to the Macro-1 cell cluster. (**B**) Monocle trajectory inference places 3 myeloid cell clusters (Mono, Macro-1, and Macro-2) at discrete nodes, and cDC and Macro/Mono cell clusters are placed in the middle branch, indicating their transitional states. (**C**) Monocle pseudotime inference traces a path from the Mono cell cluster node to the Macro-1 cell cluster node.

In focusing our attention on kidney allograft–infiltrating myeloid cells, we performed RNA Velocity on the identified myeloid cell clusters. As shown in Figure 4A, the velocity vectors among the myeloid cell clusters in the kidney allograft traced a putative differentiation path from monocytes (green) to macrophages (red). Here, the RNA Velocity analysis was performed on pooled cells from all conditions as in Figure 1A. However, data in Figure 2 clearly demonstrate that the same cell populations from rejecting versus tolerized kidneys manifested significant differences in their gene expressions, suggesting their distinct function under different conditions. To complement our findings from RNA Velocity analysis, we also performed a second trajectory analysis on the same myeloid cell clusters using Monocle (46). First, based on their DEGs, Monocle clustered and placed myeloid cells similar to Seurat (Figure 4B and Figure 1A), indicating that cell clustering earlier by Seurat was robust. Next, using Monocle’s clustering, we placed the myeloid cell clusters along a trajectory of pseudotime (Figure 4C) and found that the result replicated that by RNA Velocity analysis (Figure 4A). Specifically, the trajectory began at the Mono cell node (darkest blue) and ended at the Macro-1 and Macro-2 nodes (lightest blue, Figure 4C). The middle branch of the trajectory consisted mostly of conventional DC (cDC) and Macro/Mono cells, indicating that these populations represented transitional cell states in this particular differentiation path. Of note, in using the Monocle tool, we first confirmed that cells from all different samples showed a similar distribution on the pseudotime plot (data not shown), which ensured that our observed cell differentiation trajectory was not a result of sample-to-sample variability. Collectively, our data from RNA Velocity and Monocle pseudotime led to a similar conclusion that intra-graft myeloid cells differentiate from monocytes to macrophages in the context of kidney transplantation.

### Axl is differentially expressed by graft-infiltrating macrophages in rejecting versus tolerized kidney allografts.

**Figure 5:**
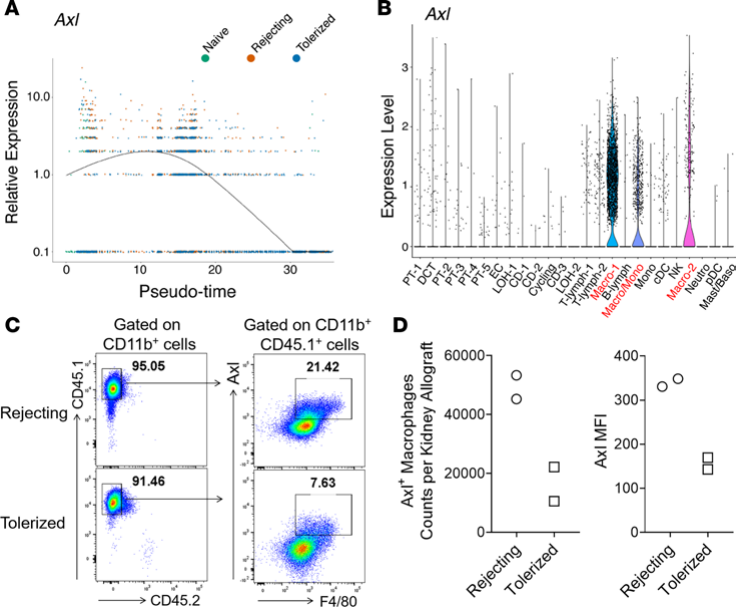
*Axl* is differentially expressed by graft-infiltrating macrophages in rejecting versus tolerized kidney allografts. (**A**) Expression of *Axl* as a function of pseudotime by myeloid cell clusters from the indicated groups. (**B**) Violin plot demonstrating that *Axl* is predominantly expressed by 3 myeloid cell clusters (Macro/Mono, Macro-1 and Macro-2) only. (**C**) CD45.2^+^ BALB/c kidneys were transplanted into congenic CD45.1^+^ WT B6 recipients. Recipients were either treated with donor ECDI-SP (tolerized) or not (rejecting). Kidney allografts were retrieved on d15 after transplantation for FACS analysis. Representative FACS plots showing intragraft CD45.1^+^ recipient–derived (majority) and CD45.2^+^ donor–derived (minimal) myeloid cells (left panels, cells were gated on total live CD11b^+^ myeloid cells, representative of *n* = 2 in each group). CD45.1^+^ recipient myeloid cells were further analyzed for their expression of Axl and F4/80 (right panels, representative of *n* = 2 in each group). (**D**) Left: total numbers of Axl^+^F4/80^+^ macrophages per kidney allograft as shown in **C**. Right: MFI of Axl expression normalized to the isotype control.

In the above pseudotime analysis, we noted that the expression of the gene *Axl* decreased as a function of pseudotime, where the terminus of negligible *Axl* expression was predominated by myeloid cells in tolerized kidneys (Figure 5A). This observation suggested that, in contrast to previous literature suggesting its role in resolution of inflammation in autoimmunity, Axl may in fact function to promote rejection in transplantation. To support the importance of Axl in myeloid cells in our kidney transplant model, we further observed that, among the top cluster-defining genes, *Axl* was exclusively expressed only by the graft-infiltrating myeloid cell clusters Macro-1, Macro-2, and Macro/Mono — and not by any other cell types (Figure 1B, Figure 5B, and Supplemental Figure 6).

To determine the role of Axl in kidney rejection, we next examined *Axl* expression at the protein level in kidney allografts. We transplanted congenic CD45.1^+^ B6 recipients with CD45.2^+^ BALB/c kidneys, and we analyzed the kidney allografts by FACS on d15 after transplantation. Based on congenic markers, we first confirmed that, on posttransplantation d15, intragraft CD11b^+^ cells in the kidney allograft were essentially entirely of recipient origin (CD45.1^+^) in both rejecting or tolerized recipients, and that Axl was predominantly expressed by F4/80^+^ macrophages in both groups (Figure 5C). Interestingly, we found that the total number of Axl^+^ macrophages, as well as their level of Axl expression, were substantially higher in rejecting kidneys in comparison with tolerized kidneys (Figure 5D). Collectively, these data indicate that *Axl* is predominantly expressed by recipient-derived graft-infiltrating macrophages after transplantation and that its elevated level of expression correlates with kidney allograft rejection.

### Axl promotes intragraft differentiation of inflammatory macrophages in kidney allografts.

**Figure 6:**
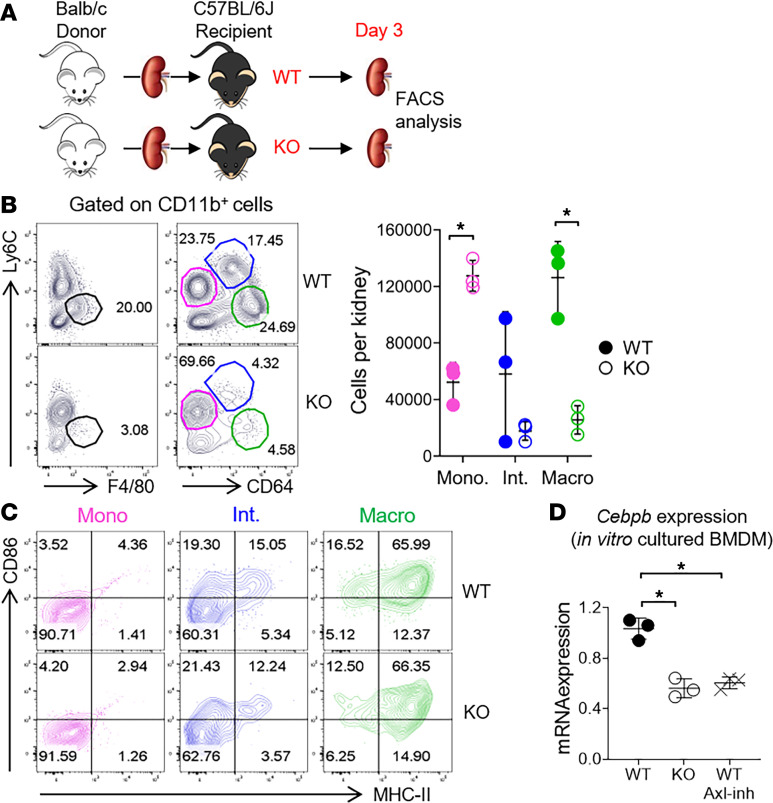
Axl promotes intragraft differentiation of inflammatory macrophages in kidney allografts. (**A**) Scheme of allogeneic kidney transplantation and graft harvest. BALB/c kidneys were transplanted into bilaterally nephrectomized *Axl* WT or -KO B6 recipients. Kidney allografts were retrieved on d3 after transplantation for FACS analysis. (**B**) Representative FACS plots showing F4/80 (left panels) and CD64 (right panels) among graft-infiltrating CD11b^+^ cells in kidney allografts from WT or KO recipients. Scatter plot showing the total number of various subsets of myeloid cells (with corresponding colors to their respective gates). *n* = 3 per group. *P < 0.05 (1-way ANOVA). (**C**) Expression of MHC II and CD86 by various subsets of myeloid cells shown in **B** (with corresponding colors to their respective gates). (**D**) Gene expression of *Cebpb* determined by qPCR in cultured BMDC from *Axl* WT and -KO mice. In WT BMDM, Axl kinase activity was further inhibited by bemcentinib (1 μM, during the last 2 days of culture). *n* = 3 per group. *P < 0.05 (1-way ANOVA).

We next investigated the role of Axl on macrophage differentiation (as suggested by RNA Velocity and Monocle trajectory in Figure 4) in kidney allografts. To do so, we took advantage of B6 mice with *Axl* gene deficiency. *Axl* WT or -KO B6 recipients were transplanted with BALB/c kidneys and sacrificed on d3 after transplantation for analysis (Figure 6A). As shown in Figure 6B, in WT recipients, a significant F4/80^+^ macrophage population was present on d3 after transplantation, but this population was markedly diminished in KO recipients. In response to injuries such as transplantation, circulating Ly6C^hi^ monocytes are known to infiltrate into the grafts and differentiate into Ly6C^lo^ macrophages after transplantation (47). To test if Axl regulated the differentiation of graft-infiltrating myeloid cells after transplantation, we phenotyped graft-infiltrating CD11b^+^ cells based on their Ly6C and CD64 (FcγR1) expressions. While Ly6C is expressed by monocytes, the expression of CD64 represents the differentiation of monocytes to inflammatory macrophages (48, 49). As shown in Figure 6B, in kidney allografts from WT recipients, 2 prominent cell populations, CD64^+^Ly6C^hi^ and CD64^+^Ly6C^lo^ cells, were identified in addition to CD64^–^Ly6C^+^ cells, which represented monocytes. The CD64^+^Ly6C^lo^ cells expressed F4/80 (data not shown) and downregulated Ly6C, therefore representing a true macrophage population, whereas the CD64^+^Ly6C^hi^ cells likely represented an intermediate phenotype between monocytes and macrophages. Interestingly, in contrast to kidney allografts from WT recipients, those from KO recipients had a drastically reduced number of both CD64^+^Ly6C^hi^ and CD64^+^Ly6C^lo^ cells. The scatter plot on the right depicted the total numbers of cells of the 3 myeloid cell subpopulations in WT versus KO recipients (CD64^–^Ly6C^+^ monocytes, CD64^+^Ly6C^hi^ intermediate cells, and CD64^+^Ly6C^lo^ macrophages). We further examined the expression of MHC II and CD86 by these populations. As shown in Figure 6C, in CD64^–^Ly6C^+^ monocytes, the expression of both MHC II and CD86 was minimal (pink), whereas in CD64^+^Ly6C^lo^ macrophages (green), their expression was at the highest levels. In CD64^+^Ly6C^hi^ intermediate cells (blue), their expression was at intermediate levels. The pattern of increased expression of both MHC II and CD86 further supported an Axl-dependent intragraft differentiation of inflammatory macrophages from the less inflammatory monocytes as early as d3 after transplantation.

To further investigate potential signaling pathways implicated in Axl-mediated monocyte differentiation, we examined CCAAT/enhancer-binding protein β (C/EBPβ). C/EBPβ is a member of the C/EBP family of transcription factors known to be highly expressed in monocytes and macrophages to regulate the expression of target genes involved in their differentiation (50). As shown in Figure 6D, we measured the expression of *Cebpb* in BMDM generated from *Axl* WT and KO mice, and we found that the expression of *Cebpb* was significantly downregulated in KO BMDM in comparison with that in WT BMDM. We further confirmed that, in the absence of Axl, in vitro macrophage differentiation from BM precursors, as evidenced by their enhanced CD64 expression, was also impaired (Supplemental Figure 7). Interestingly, pharmacological inhibition of the kinase activity of Axl by an Axl inhibitor bemcentinib (51) suppressed *Cebpb* expression in WT BMDM to that in KO BMDM (Figure 6D). These in vitro data, thus, support the in vivo finding that monocyte differentiation is impaired in the absence of Axl (Figure 6B).

### Axl promotes T cell recruitment and activation in kidney allografts.

**Figure 7:**
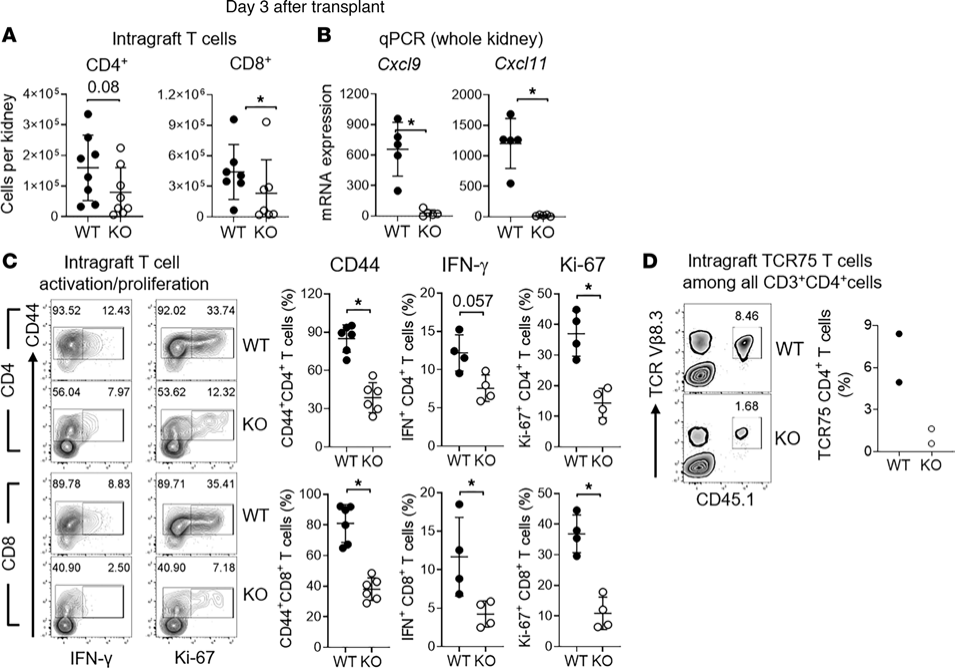
Axl promotes T cell recruitment and activation in kidney allografts. (**A**) Scatter plots of the total number of graft-infiltrating CD4^+^ and CD8^+^ T cells in *Axl* WT or -KO recipients on d3 after transplantation (*n* = 7–8 per group). *P < 0.05 (*t* test). (**B**) Expression of chemokines *Cxcl9* and *Cxcl11* measured by qPCR in whole kidney allografts retrieved on d3 (*n* = 5 per group). *P < 0.05 (*t* test). (**C**) Representative FACS plots showing cell activation (CD44), effector function (IFN-γ), and proliferation (Ki-67) of graft-infiltrating CD4^+^ and CD8^+^ T cells in *Axl* WT or -KO recipients. Scatter plots showing statistical comparisons between the groups. *n* = 4 per group. *P < 0.05 (*t* test). (**D**) Purified alloantigen-specific TCR75 CD4^+^ T cells (2.5 × 10^5^ per mouse) were adoptively transferred into *Axl* WT and -KO recipients 1 day prior to transplantation. Representative FACS plots demonstrating intragraft TCR75 CD4^+^ T cells in *Axl* WT and -KO recipients on d3 after transplantation. TCR75 CD4^+^ T cells were identified by CD45.1 and TCR Vβ8.3. Cells were gated on total CD3^+^CD4^+^ T cells (*n* = 2 per group). Scatter plot demonstrating percentage of TCR75 cells among total CD3^+^CD4^+^ T cells in each group.

We next determined intragraft infiltration of T cells in both WT and KO recipients on d3 after transplantation. As shown in Figure 7A, total graft-infiltrating CD4^+^ and CD8^+^ T cells were notably reduced in KO recipients in comparison with WT recipients. As graft T cell recruitment is mediated by interactions between chemokines CXCL9/CXCL11 and their cognate receptor CXCR3 on T cells (52), we next examined the expression of these molecules in the kidney allograft. We found that the expression of CXCR3 on T cells (by FACS) was comparable in WT or KO recipients (data not shown). However, the expression of *Cxcl9* and *Cxcl11* (by qPCR) was significantly reduced in KO recipients in comparison with WT recipients (Figure 7B), suggesting that Axl plays a critical role in promoting local chemokine expressions and intragraft T cell recruitment.

Next, we determined if graft-infiltrating T cells were less activated and/or proliferative in KO recipients due to the fewer graft-infiltrating inflammatory macrophages expressing high CD86 and MHC II (Figure 6C). As shown in Figure 7C, a significantly lower percentage of CD4^+^ and CD8^+^ T cells in KO recipients expressed the activation marker CD44 than in WT recipients. Furthermore, IFN-γ– and Ki-67–expressing cells were also markedly fewer in both CD4^+^ and CD8^+^ T cells in KO recipients in comparison with WT recipients (Figure 7C, scatter plots), demonstrating their impaired effector function and proliferation.

In addition to total graft-infiltrating T cells, we also examined the effect of *Axl* deficiency on donor-specific T cells. Here, we took advantage of the CD45.1^+^ TCR transgenic CD4^+^ T cells from TCR75 mice, which recognize a BALB/c K^d^ peptide presented by the B6 MHC II molecule I-A^b^ (53). Purified TCR75 CD4^+^ T cells were adoptively transferred into WT or KO B6 recipients 1 day prior to transplantation and examined in kidney allografts similarly on d3 after transplantation. As shown in Figure 7D, KO recipients harbored a significantly lower number (~7-fold) of alloantigen-specific TCR75 CD4^+^ T cells in comparison with WT recipients. Collectively, these data suggest that, in allogeneic kidney transplantation, recipient myeloid cell *Axl* expression promotes CD4^+^ and CD8^+^ T cell graft infiltration, their intragraft activation, and their proliferation.

### Targeting Axl reduces kidney allograft inflammation.

**Figure 8:**
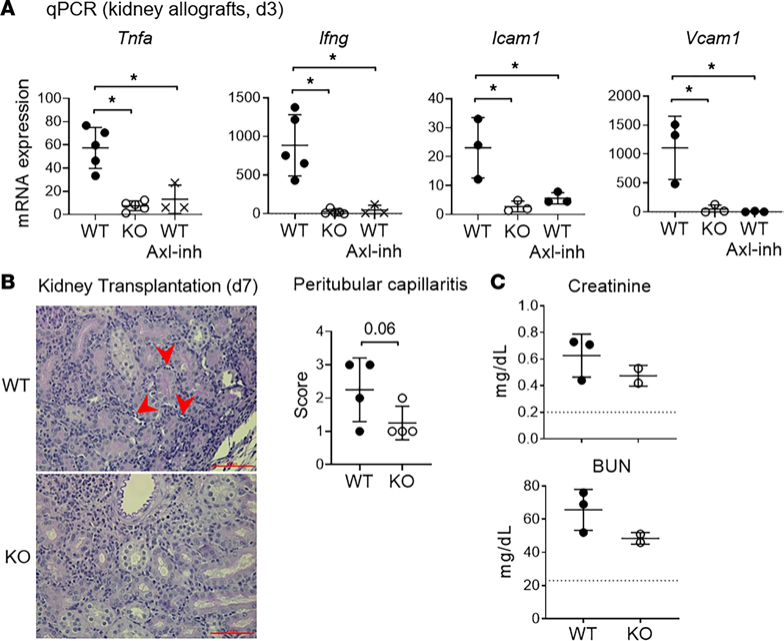
Targeting Axl reduces kidney allograft inflammation. (**A**) Scatter plots showing the expression of inflammatory cytokines and cell adhesion molecules measured by qPCR in kidney allografts from indicated recipients on d3 after transplantation (*n* = 3–5 per group). *P < 0.05 (1-way ANOVA). (**B**) Representative photomicrograph of histopathology of kidney allografts from *Axl* WT and -KO recipients retrieved on d7 after transplantation. Red arrowheads point to peritubular capillaritis. Scale bar: 100 μm. Scatter plot depicting histopathologic scores for kidney allograft peritubular capillaritis from *Axl* WT and -KO recipients (*n* = 4 per group). *P* value was calculated by 1-tailed Welch’s *t* test. (**C**) Measurement of serum creatinine and blood urea nitrogen (BUN) on d7 after transplantation. Dotted lines indicate the average normal values in naive B6 mice (*n* = 2–3 per group).

Early macrophage graft infiltration and graft inflammation are associated with poor kidney transplant outcomes (54). Data in Figure 6B reveal a significant reduction in early (d3) macrophage graft infiltration in *Axl*-KO recipients in comparison with WT recipients. We next investigated if recipient *Axl* deficiency also reduced early intragraft inflammation. As shown in Figure 8A, proinflammatory cytokines *Tnfa* and *Ifng* were significantly reduced in kidney allografts retrieved from *Axl*-KO recipients in comparison with WT recipients on d3. Additionally, the expression of *Icam1* and *Vcam1,* 2 target genes of TNF-α, was also reduced significantly in kidney allografts from KO recipients. Furthermore, such reductions seen in *Axl*-KO recipients were effectively recapitulated by a short peritransplant (d–1 to d+3) recipient treatment with the Axl inhibitor bemcentinib (Figure 8A). We next examined the histopathology of kidney allografts from WT or KO recipients at a slightly later time point on d7 after transplantation. Consistent with lower levels of early (d3) proinflammatory cytokines and adhesion molecules shown in Figure 8A, allografts on d7 from KO recipients exhibited a lower score of peritubular capillaritis in comparison with WT recipients (Figure 8B). We also observed a trend for lower serum levels of creatinine and BUN (Figure 8C) in KO recipients in comparison with WT recipients. However, at this early time point, these differences did not reach statistical significance, indicating that during the early posttransplantation period, conventional serum biochemical parameters of kidney function are not sufficiently sensitive to detect allograft injury in this mouse model of kidney transplantation.

## Discussion

The present study employed an unbiased transcriptomics approach at a single cell level for examining kidney allograft–infiltrating myeloid cells and identified a potentially novel recipient macrophage Axl-dependent pathway that plays a critical role in acute rejection in kidney transplantation. While its roles in inflammation and autoimmunity have been previously demonstrated, this is the first study to our knowledge of the role of Axl in transplant alloimmunity. Furthermore, in contrast to previous literature describing an aspect of Axl in inhibiting inflammation (26), the present study points to its function in promoting the differentiation of inflammatory graft-infiltrating macrophages and subsequent intragraft donor-specific T cell immune responses, therefore joining the very few publications describing its priming role in T cell activation.

Signaling partners of Axl in a given cellular environment ultimately dictate the consequence of Axl activation in that cell. For instance, in cancer cells, coactivation of Axl with EGFR (55) or CUB domain–containing protein-1 (CDCP1) (56) has been shown to enhance cancer cell survival and their invasiveness. This interaction is also known to promote the expression of immune checkpoint molecules such as programmed death-ligand 1 (PD-L1) (57) to blunt anti-\tumor immunity. On the other hand, coactivation of Axl with TLRs and/or inflammasomes in macrophages may lead to their production of inflammatory mediators that support T cell priming (58). Our current study identifies a downstream transcription factor Cebpb as a potential mediator of the Axl-dependent monocyte differentiation (Figure 6D). Combined with published literature demonstrating potential cross-talks between Cebpb and TLR pathways (59) or mTOR pathways (60), our study, thus, supports an Axl-initiated signaling cascade implicated in macrophage differentiation and polarization. It further supports future investigations of Axl as a potential target for modulating macrophage behavior in response to inflammation. In this context, investigations of the role of myeloid cell Axl in solid organ transplantation models other than kidney transplantation, as well as the utility of clinically translatable Axl inhibitors such as bemcentinib in promoting long-term allograft function in these models, would be highly informative and are currently under active investigation. It is important to acknowledge, however, that it remains unknown at this junction whether Axl, as a member of the cell surface RTK family TAM, is also involved in the induction of transplant tolerance, as is the case for another member of the same family, Mertk (61).

Regardless of its signaling partners and cellular effects, a common feature of Axl ligation is the proteolytic cleavage of cell-surface Axl to generate soluble Axl that can be detected in circulation (62). Our study, therefore, also supports the utility of quantifying circulatory soluble Axl as a marker for early posttransplant graft injury and/or for predicting the magnitude of future alloimmunity. These possibilities warrant future studies in animal models and in human transplant recipients.

One of the most striking findings revealed by the present study is the identification of a ribosomal protein gene signature (*Rpl41*, *Rpl38* and *Rplp1* belong to the 60S subunit; *Rps28*, *Rps27*, *Rps26*, *Rps21*, and *Fau* belong to the 40S subunit) distinctly expressed in major myeloid cell clusters in tolerized kidneys in comparison with rejecting kidneys (Figure 2). The expression of ribosomal protein genes and ribosome biogenesis are highly energy-consuming cellular processes tightly regulated in response to intracellular and environmental stimuli (63). Consistently, we observed upregulation of several genes (e.g., *Usmg5*, *Aldob*) involved in energy production in these cell clusters from tolerized kidneys (Supplemental Figure 8). These data, therefore, support that tolerance is an active process involving increased expression of ribosomal protein genes and ribosomal biogenesis, particularly in graft-infiltrating myeloid cells.

Under homoeostatic conditions, various ribosomal proteins are synthesized stoichiometrically with rRNA (18S, 28S, 5.8S, and 5S) to produce equimolar amounts for ribosomal biogenesis. However, under unique physiological (e.g., growth and differentiation) and pathological (e.g., cancer) conditions, the expression levels of specific ribosomal protein genes are altered (64–66). Several studies have shown that the expression of ribosomal genes is downregulated during myogenesis, osteogenesis, adipogenesis, granulopoiesis, and monocytic differentiation (67–70). Interestingly, inhibition of ribosomal genes by actinomycin D promotes the differentiation of myeloid cells from hematopoietic stem cells (71) and triggers activation of NLRP3 inflammasome in macrophages, causing their production of the inflammatory cytokine IL-1β (72, 73). Furthermore, macrophages depleted of a ribosomal protein L13a attain inflammatory phenotype and promote tissue injuries in several murine models of inflammatory diseases (74–76). In fact, L13a can specifically bind to mRNA transcripts of inflammatory cytokines and silence their translation, thus imparting an antiinflammatory function. These data support that expressions of ribosomal genes and ribosome biogenesis are tightly linked to macrophage differentiation and their function. Data from our current study (Figure 2) further support that induction of transplant tolerance may, in part, depend on altering myeloid cell differentiation and their function by upregulating expressions of ribosomal protein genes. This area warrants further investigations with the same degree of validation placed on Axl as we did in this manuscript.

So far, only limited information on the use scRNA-seq in transplantation has been published (77). Interestingly, in a scRNA-seq study of a kidney allograft biopsy from a recipient undergoing acute rejection, distinct myeloid populations were also identified (22), similar to our findings here, therefore independently supporting a role of these cells in kidney rejection in humans. Future studies of such cell populations in transplant patients with or without graft rejection, with different types (antibody-mediated versus cell-mediated) or severity (Banff grades) of rejections would be of significant importance to our understanding of the role of these cells in allograft inflammation. Our current study has provided a scientific rationale and a mechanistic basis for such future studies. In addition to *Axl*, the data set presented in the current study also implicated additional processes and gene networks previously poorly understood in allograft rejection or protection. We recognize that an intrinsic limitation of studies involving scRNA-seq is the experimental artifacts introduced by variable cell survival during cell elution from a solid matrix such as the kidney. However, such systematic artifacts were minimized in our study by simultaneous and identical processing of kidney samples from various groups (Supplemental Figure 1), thereby allowing differences identified by comparison between groups to be the most meaningful. Ultimately, such unbiased studies are expected to reveal new mechanistic insights on transplant immunity that will guide our discovery of potentially novel biomarkers of allograft health and therapeutics for promoting allograft survival.

## Methods

### Mice.

Male CD45.2 and congenic CD45.1 C57BL/6 (B6 WT; I-A^b^) mice were purchased from The Jackson Laboratory. BALB/c (I-A^d^), Axl-KO (B6 background), and TCR transgenic TCR75 mice (B6 background) mice were bred in the specific pathogen–free facility at Duke University. The TCR75 CD4 T cells recognize an allopeptide (Kd peptide sequence 54–68; K^d^ 54–68) from the BALB/c MHC I molecule H2-K^d^ presented by the B6 MHC II molecule I-A^b^ (78). All genetically modified strains of mice used in the current study underwent greater than 10 generations of backcrossing.

### Mouse orthotopic kidney transplantation, transplant tolerance induction by ECDI-SP, and recipient treatment with the Axl inhibitor bemcentinib.

Kidneys from 6- to 7-week-old BALB/c (male or female) mice were transplanted into bilaterally nephrectomized B6 (WT or KO) male recipients. In some of the experiments, kidney allograft tolerance was induced in the recipients by infusions of donor apoptotic cells. In brief, BALB/c SP were treated with ECDI (Calbiochem, 150 mg/mL per 3.2 × 10^8^) on ice for 1 hour with agitation and washed. A total of 1 × 10^8^ BALB/c ECDI-SP cells was infused to the B6 recipient i.v. on d–7 and d+1, while d0 was designated as the day of transplantation (27). In additional experiments, WT B6 recipients were further treated with bemcentinib, a selective inhibitor of Axl kinase activity (MedChemExpress). Recipients received daily oral gavage of bemcentinib (100 mg/kg/day) from d–1 to d+3. To investigate donor antigen–specific T cell responses, 2.5 × 10^5^ purified TCR75 CD4^+^ T cells were adoptively transferred into WT or KO B6 recipients 1 day prior to the first infusion of donor apoptotic cells. In some of the experiments, congenic CD45.1 B6 mice were used as recipients for kidneys from CD45.2 BALB/c donors.

### Preparation of single cells and cDNA libraries, as well as sequencing.

Kidney allografts from untreated and tolerized WT B6 recipients were retrieved on d15 after transplant, perfused by cold PBS, and a single cell suspension was prepared using a Multi Tissue Dissociation Kit2 (Miltenyi Biotec) as per manufacturer’s protocol. Naive B6 kidneys retrieved from WT B6 mice were similarly processed to serve as a control. Cell suspension was subjected to RBC lysis followed by washing. Cell viability was confirmed to be > 90%, and cell concentration was adjusted to 2000 cells/μL. The resulting single cell preparation (~10,000 cells/sample) was subjected to 10× Chromium Single Cell Instrument (10× Genomics) for barcoding and cDNA library construction. In brief, 10× Genomics microfluidic platform combines single cells with barcoded gel beads containing essential components for cDNA preparation, including barcodes to tag individual transcripts from the cells, a UMI to identify PCR products, and primers for amplification of resulting cDNA. cDNA libraries were checked for quality using Agilent D5000 Tape Station analyzer. cDNA libraries of approximately 400 bp in size were subjected to an Illumina NextSeq 550 sequencer using NextSeq 500/550 High Output Kit v2.5 (150 Cycles).

### scRNA-seq data processing.

10× Genomics–generated reads were converted to fastq, aligned to the mm10 reference genome, filtered, and counted using 10× Genomics’ CellRanger pipeline. Reads were used to construct individual Seurat objects for each sample following filtering on a per-sample basis to eliminate cells with low or exceedingly high UMI counts, or those with high ratios of mitochondrial gene expression. Doublets were removed using DoubletDecon. After filtering, all objects were integrated using the SCTransform integration workflow on Seurat. Clustering, marker gene identification, and differential expression analyses were all performed using Seurat. Cluster marker genes were identified using FindConservedMarkers function. DEGs were identified through FindMarkers function using DESeq2. A full data set UMAP was generated using Seurat’s DimPlot function. All heat maps were generated using Seurat’s DoHeatmap plotting function, using scaled data in the RNA assay as input data for the specific gene expression. Dot plots were generated using the DotPlot plotting function in Seurat, with normalized counts in the RNA assay as input data. Violin plots were generated using Seurat VlnPlot plotting function, using normalized counts in the RNA assay as input data. Representative UMAPs were generated using Seurat’s FeaturePlot plotting function, using normalized counts in the RNA assay as input data.

### DEG analysis.

A pseudobulk approach was used to identify differential genes between the tolerized and rejecting samples in Macro-1 or Macro/Mono clusters. For the Macro-1 cluster, pseudobulk samples were created by pooling raw read counts across Macro-1 cells for each tolerized or rejecting sample. Differential analysis was performed with DESeq2 (79), using the pseudobulk samples. *P* values and normalized read counts were obtained from DESeq2 output. FDRs were obtained from *P* values using the Benjamini-Hochberg procedure to minimize false positive results. Genes with FDR < 0.05 were considered significant. The same procedure was used to identify differential genes for the Macro/Mono cluster. A similar approach was also used to identify differential genes between cells in Macro clusters and cells in other clusters (Supplemental Figure 6). Specifically, pseudobulk samples for Macro clusters were created by pooling raw read counts across Macro-1, Macro-2, and Macro/Mono cells for each sample (naive, rejecting, or tolerized). Pseudobulk samples for other clusters were created likewise by pooling cells from clusters other than the 3 Macro clusters. DESeq2 was performed to compare pseudobulk samples for Macro clusters and other clusters. Dummy variables indicating which sample each pseudobulk was created from were added in the DESeq2 model. FDR and normalized expression values were obtained from DESeq2 output as described above.

### LR analysis.

Single cell gene expression data and clustering information from the final integrated Seurat object were used as input for cell_signaling function using SingleCellSignalR. LR data were generated using cell_signaling function. LR interaction network diagrams were generated using visualize function in SingleCellSignalR.

### RNA velocity.

Loom files generated by command-line tool Velocyto from the original CellRanger reads were combined using loompy combine function in Python3 using the “Accession” key. Data were subsetted to only contain cells from myeloid cell clusters. Velocity estimates were made using Seurat’s RunVelocity function. RNA Velocity Plot was generated using Velocyto.R’s show.velocity.on.embedding.cor function.

### Monocle.

Myeloid cell clusters (Macro-1, Macro-2, Macro/Mono, Mono, and cDC) and accompanying nonnormalized gene expression count data from the final integrated Seurat object were used as inputs to create Monocle v2 newCellDataSet. Differential expression between clusters was calculated using differentialGeneTest function in Monocle. DDRTree method was used for dimensionality reduction, and the pseudotime trajectory plot was generated using Monocle’s plot_cell_trajectory function, where cells were colored by pseudotime placement. Axl expression by pseudotime was plotted using plot_genes_in_pseudotime function in Monocle.

### Data availability.

Raw scRNA-seq data (fastq files) and processed UMI count matrices used in the current study are publicly available on NCBI GEO (GSE157292).

### Histology.

Kidney allografts were fixed in 10% formalin for 10–12 hours and 5 μm–thick sections were stained for periodic acid–Schiff (PAS) staining using kit (Sigma-Aldrich) as per manufacturer’s instructions. PAS-stained kidney allograft sections were blindly evaluated for features of kidney rejection. The severity of inflammation was scored on kidney sections as 0 (none), 1 (minimal), 2 (mild), 3 (moderate), to 4 (severe).

### Kidney allograft function analysis.

Renal function was assessed by measuring serum creatinine and urea nitrogen levels by kits from Alfa Wassermann Inc. per the manufacturer’s protocols.

### Flow cytometry.

Recipients were euthanized, and kidney allografts were retrieved, perfused with cold PBS, and digested using Multi Tissue Dissociation Kit1 (Miltenyi Biotec) for single cell preparation. Cell preparations were subjected to RBC lysis and stained for surface and intracellular markers for flow cytometric analysis. For intracellular staining, cells were first stimulated with the PMA and ionomycin cocktail (eBioscience) for 4 hours at 37°C, followed by surface staining, fixation/permeabilization (eBioscience), and intracellular staining for IFN-γ (clone XMG1.2, Tonbo) and Ki-67 (clone B56, BD Biosciences). The following antibodies were used for cell surface staining: Axl (clone 175128, BD Biosciences,), CD3 (clone 17A2, eBioscience), CD4 (clone GK1.5, BD Biosciences), CD8 (clone 53-6.7, eBioscience), CD11b (clone M1/70, BD Biosciences), CD44 (clone IM7, eBioscience), CD45.1 (clone A20, BD Biosciences), CD45.2 (clone 104, BD Biosciences), CD64 (clone X54-5/7.1, eBioscience), CD86 (clone GL1, BD Biosciences), CXCR3 (clone CXCR3-173, BioLengend), F4/80 (clone BM8, BioLegend), Ly6C (clone HK1.4, eBioscience), Ly6G (clone 1A8, eBioscience), MHC II-A^b^ (clone AF6-120.1, eBioscience), and TCRVβ8.3 (clone 1B3.3, BD Biosciences). Dead cells were removed by using the Aqua live/dead dye (Molecular Probes). Stained cells were acquired on BD Fortessa, and samples were analyzed using FlowJo V.10.1 (Tree Star Inc.).

### BM-derived macrophages (BMDM) culture.

BM cells were isolated from the tibia and fibula of WT and Axl-KO mice and cultured in DMEM with M-CSF (20 ng/mL) as described earlier (80). On d5, BMDM from WT mice were further treated with the Axl inhibitor bemcentinib (1 μM). BMDM were harvested on d7, RNA was extracted using TRIzol (Invitrogen), and cDNA was prepared using reverse transcriptase PCR (Applied Biosystems).

### Semiquantitative PCR.

Semiquantitative real-time PCR (ABI Prism 7500) was performed in duplicates using TaqMan master mix. The following TaqMan primers/probe were used: *Cxcl9* (Mm00434946_m1), *Cxcl11* (Mm00444662_m1), *Ifng* (Mm01168134_m1), *Tnfa* (Mm00443258_m1), *Icam1* (Mm00516023_m1), *Vcam1* (Mm00449197_m1), and *Cebpb* (Mm00843434_s1). The ΔΔCt method was used to determine RNA expression, with *Gapdh* (Mm99999915_g1) serving as the internal control. Expressions of various genes in WT and KO recipients were normalized to their expression in naive kidney samples.

### Statistics.

In non–scRNA-seq studies, data were presented as mean ± SD. Statistical analysis was performed in GraphPad Prism 7.04 (GraphPad Inc.). Data were analyzed using 2-tailed Student’s *t* tests and 1-way ANOVA to determine statistical significance. *P* < 0.05 was considered significant.

### Study approval.

All mice used and mouse studies were performed in compliance with institutional guidelines, with the approval of the Duke University Division of Laboratory Animal Resources and IACUC. All the study procedures, including kidney transplantation and drugs used, were approved under institutional protocol no. A260-18-11.

## Author contributions

AD, EBT, and XL conceptualized the study. AD, IH, and JK performed experiments. AD, NRN, IH, ZJ, LB, and JK analyzed data. AD, NRN, and XL wrote the paper. XS, EBT, and XL edited and finalized the paper.

## Supplementary Material

supplemental data

supplemental Data Set 1

## Version 1 09/24/2020

In-Press Preview

## Version 2 10/15/2020

Electronic publication
