# Supplementary material for: Single cell transcriptomics of mouse kidney transplants reveals a myeloid cell pathway for transplant rejection
Source: JCI Insight. 2020 Oct 15;5(20):e141321. doi: 10.1172/jci.insight.141321 (PMC7605544; doi:10.1172/jci.insight.141321)
Supplement: supplemental data [file jciinsight-5-141321-s231.pdf]

Supplementary Figure 1

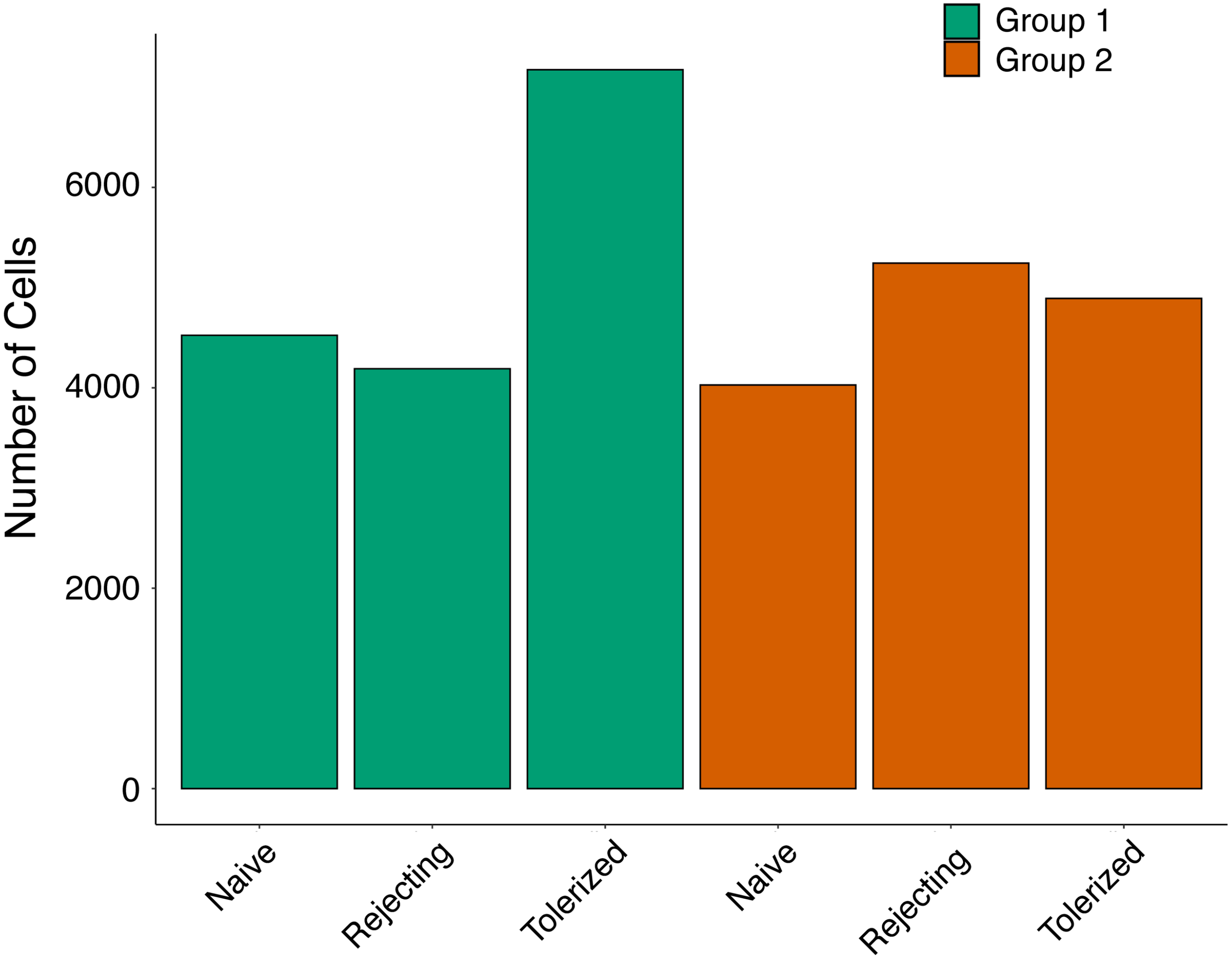

Supplementary Figure 1. The number of cells sequenced per kidney samplein each group (naïve, rejecting, tolerized), colored by the experiment number (Experiment 1 = green; and Experiment 2 = orange).

Supplementary Figure 2

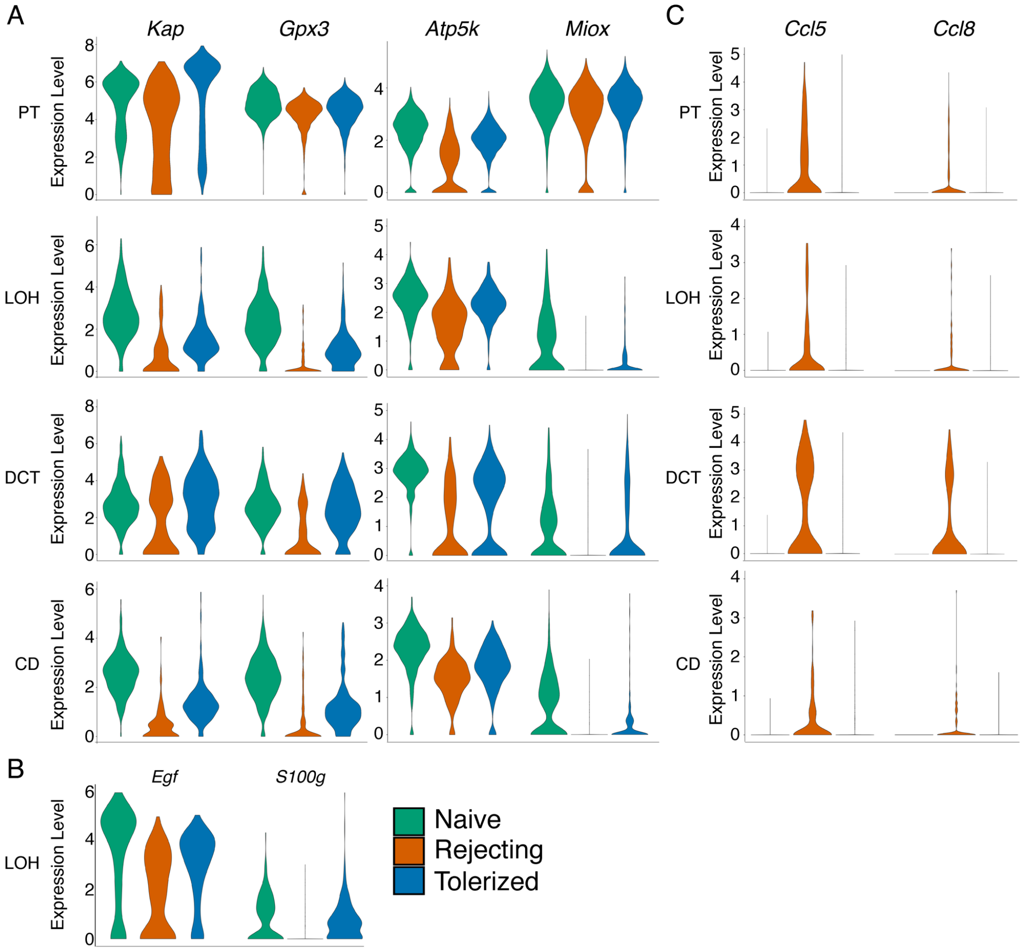

Supplementary Figure 2. Selective differential gene expression in kidney parenchymal cell clusters under various conditions reveals superior kidney function and inhibited inflammation as a hallmark of allograft tolerance. A: Violin plots depicting the expression of *Kap*, *Gpx3*, *Atp5k*, and *Miox* in various kidney parenchymal cell clusters (PT, LOH, DCT, and CD) under different conditions (naïve, rejecting, and tolerized). B: Violin plots depicting the expression of *Egf* and *S100g* in the LOH cell clusters under different conditions (naïve, rejecting, and tolerized). C: Violin plots depicting the expression of *Ccl5* and *Ccl8* in various kidney parenchymal cell clusters (PT, LOH, DCT, and CD) under different conditions (naïve, rejecting, and tolerized). PT, all proximal tubule cell clusters combined; LOH, all Loop of Henle cell clusters combined; CD, all collecting duct cell clusters combined; DCT, distal convoluted tubule cell cluster.

Supplementary Figure 3

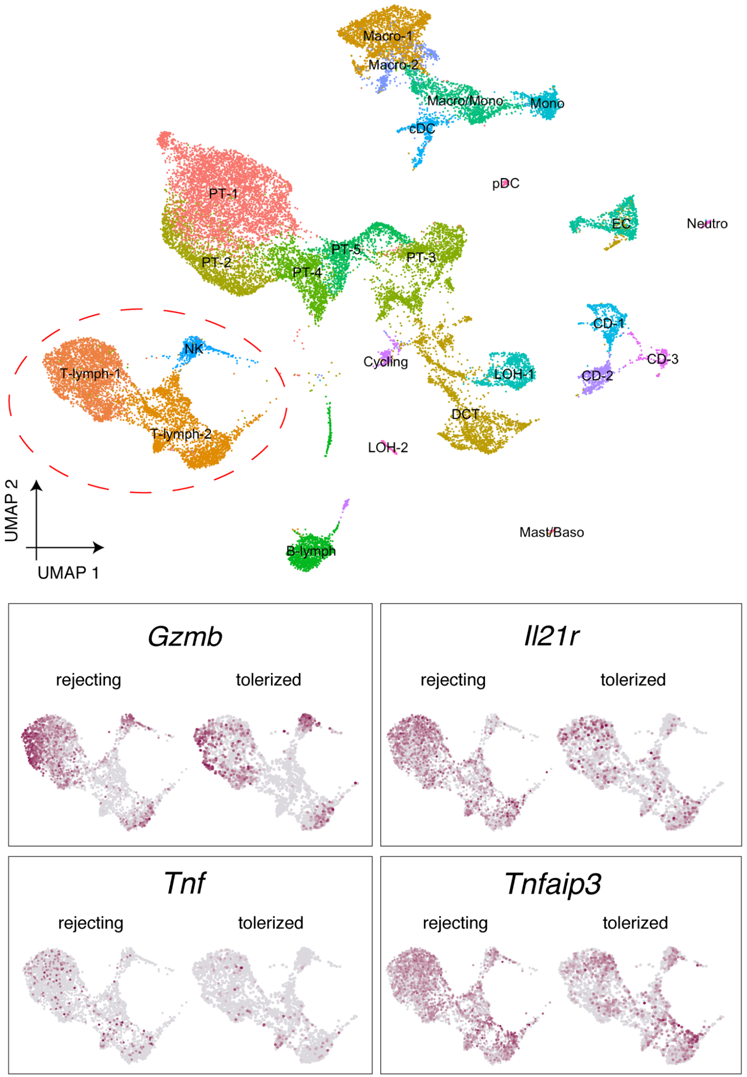

Supplementary Figure 3. DEGs in graft-infiltrating T cell clusters from rejecting versus tolerized kidney allografts. Top UMAP showing the T cell clusters (circled with the dashed line) in which the DEGs were examined. Individual UMAPs showing the expression of *Gzmb*, *Il21r*, *Tnf*, and *Tnfaip3* in T lymphocyte cell clusters (T-lymph-1 and T-lymph-2) in rejecting versus tolerized kidney allografts. T-lymph, T lymphocytes; UMAP, Uniform Manifold Approximation and Projection.

Supplementary Figure 4

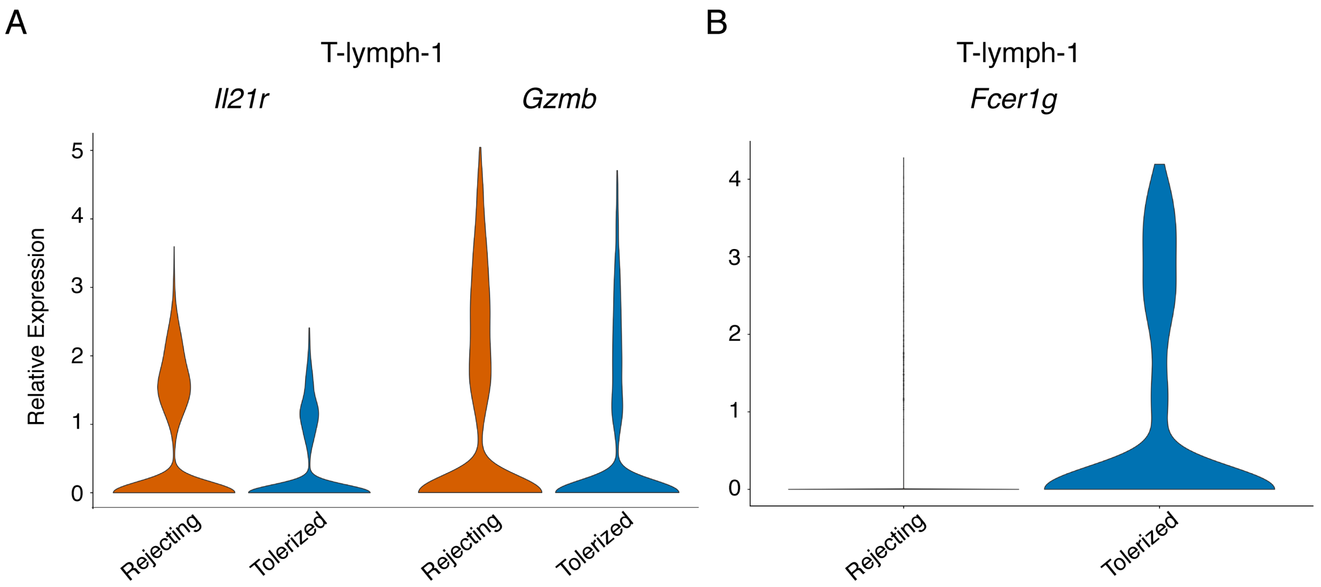

Supplementary Figure 4. Violin plots of selective differentially expressed genes in T-lymph-1 cells in rejecting versus tolerized kidney allografts. A: Violin plots depicting the expression of *Il21r* and *Gzmb* in T-lymph-1 cells from rejecting versus tolerized kidney allografts. B: The Violin plot depicting the expression of *Fcer1g* in T-lymph-1 cells from rejecting versus tolerized kidney allografts.

Supplementary Figure 5

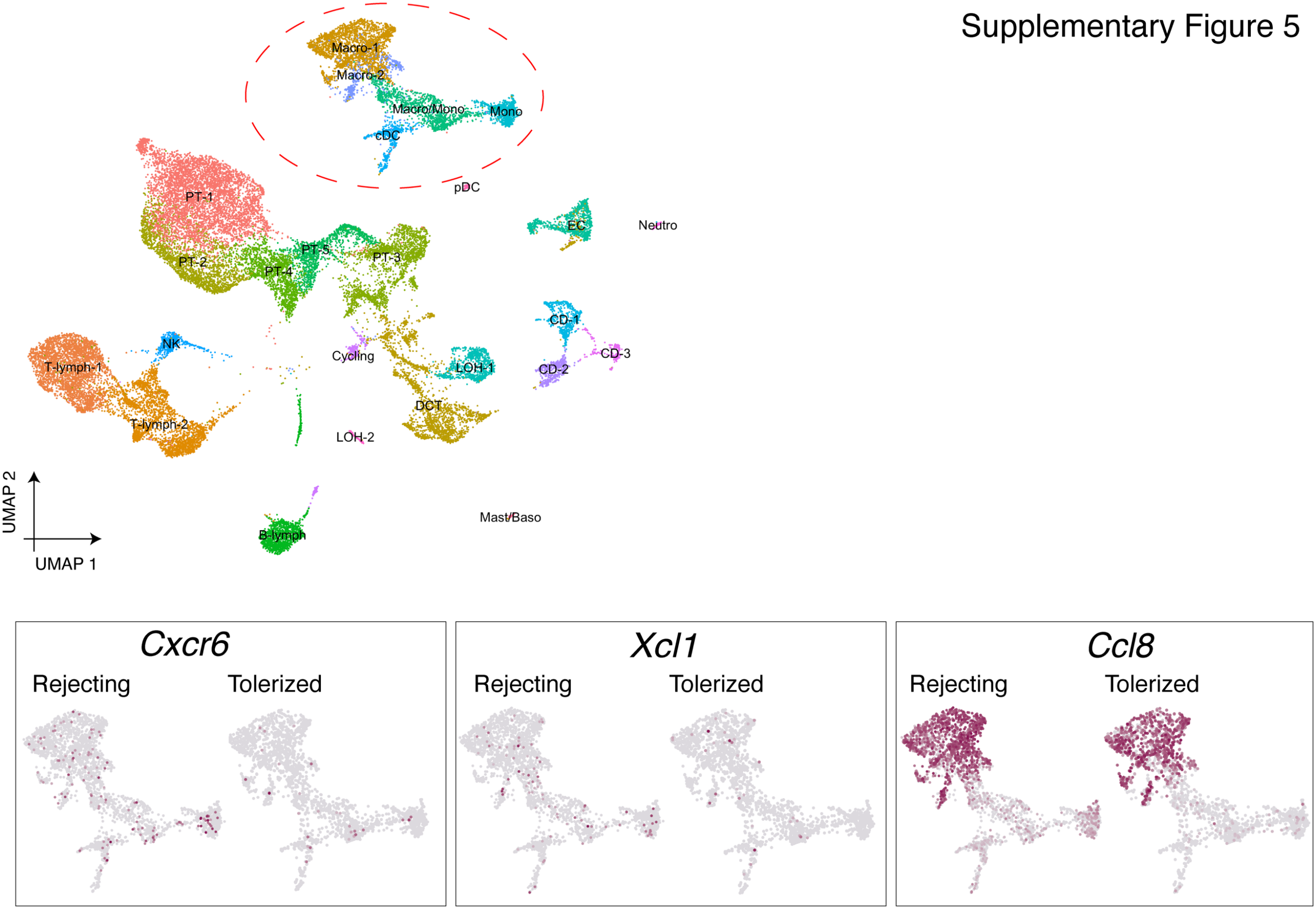

Supplementary Figure 5. DEGs in graft-infiltrating myeloid cell clusters from rejecting versus tolerized kidney allografts. Top UMAP showing the myeloid cell clusters (circled with the dashed line) in which the DEGs were examined. Individual UMAPs showing the expression of *Cxcr6*, *Xcl1*, and *Ccl8* in myeloid cell clusters (Macro-1, Macro-2, Macro/Mono, Mono, cDC) in rejecting versus tolerized kidney allografts. Macro, macrophage; Mono, monocyte; cDC, conventional dendritic cells; UMAP, Uniform Manifold Approximation and Projection.

Supplementary Figure 6

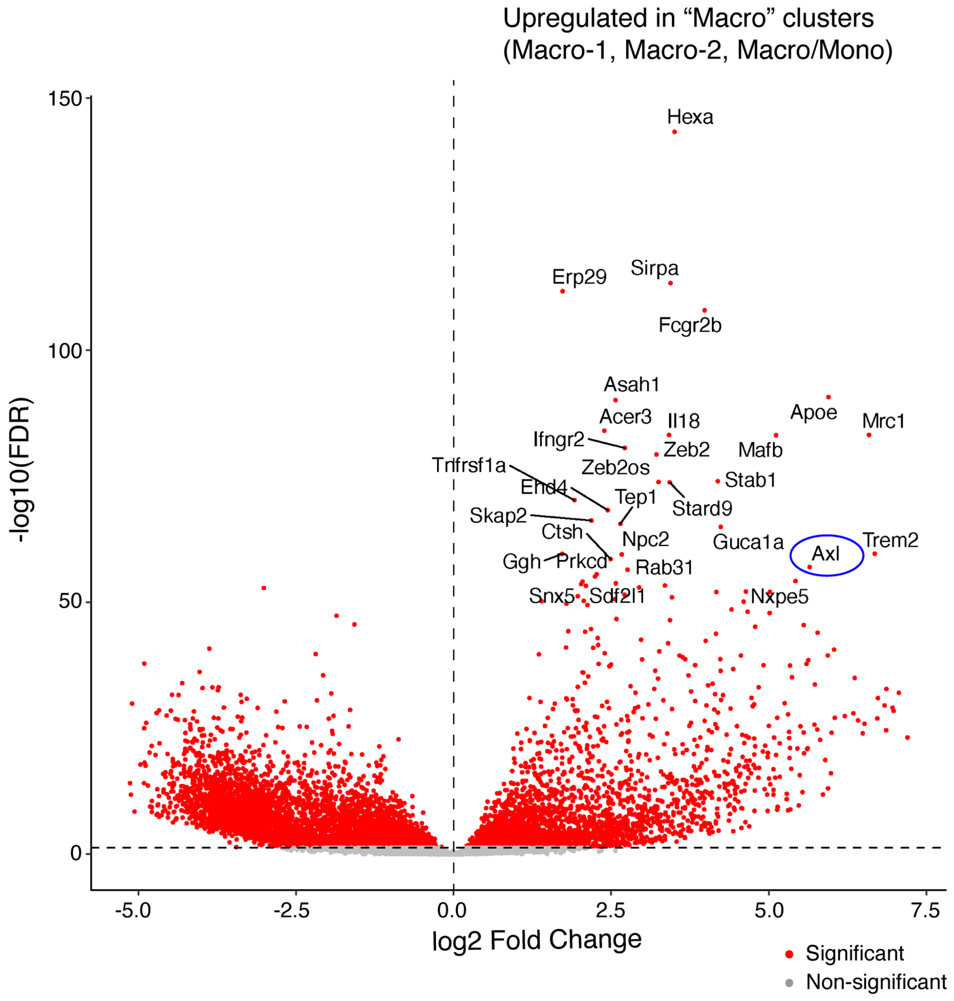

Supplementary Figure 6. Volcano plot comparing gene expressions in Macro clusters (combined Macro-1, Macro-2, Macro/Mono) and all other clusters combined across all samples (naive, rejecting, tolerized). X-axis: the log2 fold change; Y-axis: -log10(FDR). Each dot represents a gene. Significant genes (FDR < 0.05) are marked as red, and selective significant genes including Axl (circled in blue) are shown.

Supplementary Figure 7

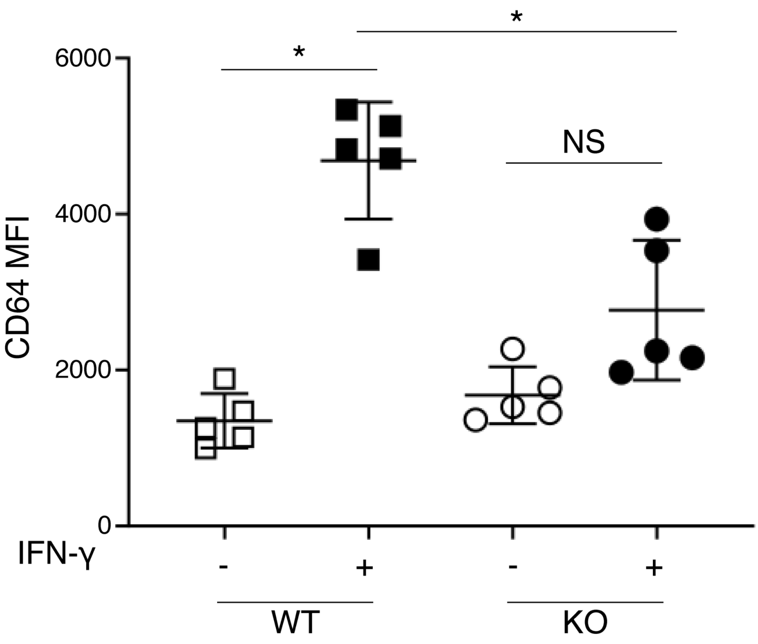

Supplementary Figure 7: Expression of CD64 by bone marrow derived macrophages (BMDM) from *Axl* WT or KO progenitors. Bone marrow progenitor cells from *Axl* WT or KO mice were isolated and cultured in medium supplemented with recombinant M-CSF (20 ng/mL) to generate BMDM. On day 7, BMDMs ($CD11b^{+}F4/80^{+}$; ~98% pure) were stimulated with recombinant IFN-γ (50 U/mL). Following 24h of stimulation, reuslting BMDMs were stained and analyzed for the expression of the inflammatory marker CD64. Scatter plot shows statistical analysis of the mean fluorescence intensity (MFI) of CD64. n = 5 preparations of BMDMs from 5 individual mice per group. *P<0.05 (one-way ANOVA); NS = Not significant.

Supplementary Figure 8

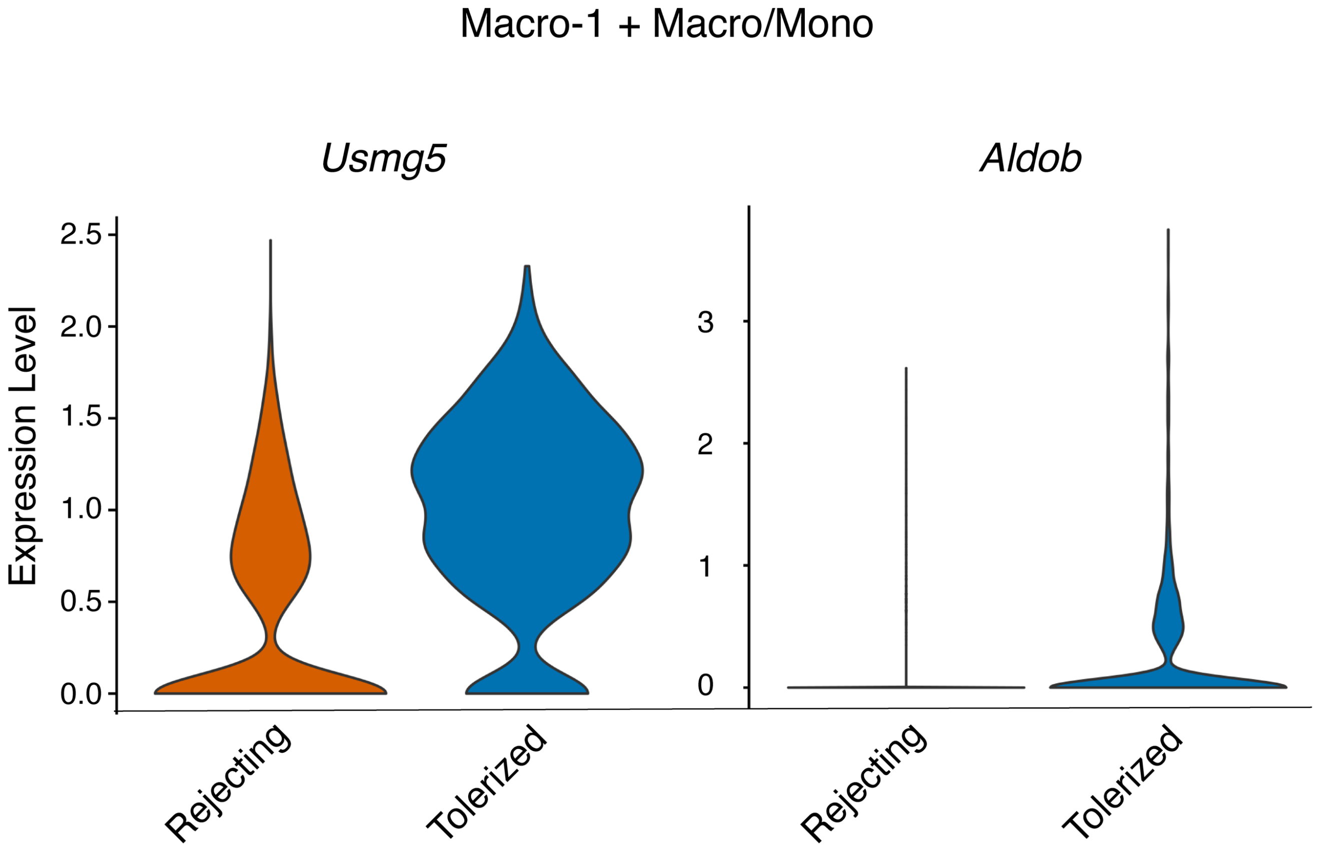

Supplementary Figure 8. Violin plots of selective differentially expressed metabolic genes in myeloid cell clusters in rejecting versus tolerized kidney allografts. Violin plots depicting the expression of genes related to metabolism and energy production (*Usmg5*, *Aldob*) in combined Macro-1 and Macro-Mono clusters from rejecting versus tolerized kidney allografts.
